# Association of the variants in the *PPARG* gene and serum lipid levels: a meta-analysis of 74 studies

**DOI:** 10.1111/jcmm.12417

**Published:** 2014-09-30

**Authors:** Qing Li, Rong Chen, Lizhan Bie, Dandan Zhao, Chunkai Huang, Jiang Hong

**Affiliations:** Department of Internal Medicine, Affiliated Shanghai First People's Hospital, Shanghai Jiao Tong UniversityShanghai, China

**Keywords:** peroxisome proliferator-activated receptorγgene, single-nucleotide polymorphisms, serum lipid level, meta-analysis

## Abstract

Considerable studies have been carried out to investigate the relationship between the polymorphisms of *PPARG* (Pro12Ala, C161T and C1431T) and serum lipid levels, but the results were inconclusive. Hence, we conducted a meta-analysis to clarify the association. MEDLINE, EMBASE and the Cochrane Library databases were searched systematically. The subgroup analysis was performed based on ethnicity. Seventy-four studies with 54,953 subjects were included in this meta-analysis. In Pro12Ala, the group with the ‘PP’ (C/C genotype) genotype group had lower levels of total cholesterol (TC) (mean difference, MD: −0.02, *P* < 0.00001; *I*^*2*^ = 28%), low-density lipoprotein cholesterol (LDL-C) (MD: −0.02, *P* < 0.00001; *I*^*2*^ = 30%) and higher levels of triglyceride (TG) (MD: 0.06, *P* < 0.00001; *I*^*2*^ = 30%) than the combined ‘PA+AA’ (PA = C/G genotype, AA = G/G genotype) genotype group in Asian population, and the group with the ‘PP’ genotype had higher levels of TG (MD: 0.07, *P* < 0.02; *I*^*2*^ = 67%) than the combined ‘PA+AA’ genotype group in non-Asian population. No statistically significant differences in the levels of TC, TG, high-density lipoprotein cholesterol, LDL-C were detected between different genotypes in C161T(Asian or non-Asian) and C1431T(Asian) polymorphisms. This meta-analysis was a renewed and confirmed study to assess the association between *PPARG* polymorphisms and serum lipid levels in Asian and non-Asian populations. There is a prominent association between Pro12Ala polymorphism and the levels of TC, LDL-C and TG in Asian population. No statistically significant differences in serum lipid levels were detected between different genotypes in C161T and C1431T polymorphisms.

## Introduction

Cardiovascular disease (CVD), a global health-threatening problem, is a complex disease resulting from many risk factors, such as genetic factor [1,2] and dyslipidaemia. Associations of different genes with predisposition to CVD have been widely examined. Disorders of lipid profile are important risk factors in CVD aetiology, and many studies have proved that serum lipid concentrations are strongly correlated with the risk of CVD, such as high levels of total cholesterol (TC) and low-density lipoprotein cholesterol (LDL-C), a low concentration of high-density lipoprotein cholesterol (HDL-C), triglyceride (TG) and apolipoprotein (Apo) B [3–6].

A large number of literatures have unravelled the correlation between genetic factors and dyslipidaemia [7–10]. Among these reported genes, the genes that have been most widely studied are the peroxisome proliferator–activated receptor (*PPAR*) genes, which have three isotypes, namely α, γ and δ. *PPARG* (as known as *PPAR*γ) is located at chromosome 3p25, and it encodes a member of the PPAR subfamily of nuclear receptors. Four *PPARG* isoforms have been identified: *PPAR*γ*1*, *PPAR*γ*2*, *PPAR*γ*3* and *PPAR*γ*4*, which result from either alternative transcription start sites or alternative splicing [11–13]. *PPARG* is potential transcriptional factors that are dietary lipid sensors [14,15]. The most common gene mutation in human *PPARG* is cytosine–guanine exchange in exon B (codon12), which results in proline to alanine substitution in the protein. Several single-nucleotide polymorphisms (SNPs) in the *PPARG* have been reported to be associated with dyslipidaemia and CVD. These SNPs include Pro12Ala (rs1801282) [13,16–29], C161T and C1431T (rs3856806) [15,17,30–35].

Various studies have investigated the association between these genetic variants and serum lipid levels in different races. However, the results were inconclusive [17,18,36–39], possibly because of the relatively small sample sizes of the individual studies or different populations have different genetic backgrounds. Therefore, we performed a meta-analysis separated from the subgroups of Asian and non-Asian populations by combining comparable published studies, leading to increased sample size and improved statistical power, to derive a more precise estimation of these associations [40].

## Materials and methods

### Search strategy

All studies reporting associations between the polymorphisms of *PPARG* (Pro12Ala, C161T and C1431T) and serum lipid levels published in English before May 2014 were identified by comprehensive computer-based searches of MEDLINE *via* PubMed, EMBASE, the Cochrane Library database and Web of Science. The following key words were used: ‘peroxisome proliferator-activated receptor γ’, ‘*PPAR*γ or PPARG gene’, ‘Pro12Ala’, ‘C161T’, ‘C1431T’, ‘polymorphism’, ‘dyslipidemia’ and ‘serum lipid level’. The search strategy described was used to obtain titles and abstracts of studies of potential relevance for this meta-analysis. The titles and abstracts were screened independently by two authors (Q Li and R Chen), who discarded studies that were not applicable (Studies were discarded when (*i*) it was not possible to extract data from either the published results or by contacting the authors or (*ii*) appropriate outcomes were not reported). For multiple reports involving the same patients, only the study with the most complete data set was included in the meta-analysis. However, for patients included in two articles, where these had different types of data of outcomes, both were included. Any disagreements about article inclusion were arbitrated by discussion with a separate reviewer (J Hong).

### Included and excluded studies

The five investigators (Q Li, R Chen, L-Z Bie, D-D zhao and C-K Huang) independently reviewed all studies identified by the search strategy, to determine whether an individual study was eligible for inclusion. The selection criteria for studies to be considered for this meta-analysis were as follows: (*i*) case–control studies published in peer-reviewed journals with full text available in English; (*ii*) studies on the relationship between *PPARG* Pro12Ala, C161T and C1431T polymorphisms and serum lipid levels; (*iii*) reporting at least one relevant outcomes of association between genotype and serum lipid levels (including TC, HDL-C, LDL-C and TG). Studies were excluded when (*i*) it was not possible to extract data from either the published results or by contacting the authors or (*ii*) appropriate outcomes were not reported.

### Types of outcome measures

(*i*) Relationship between serum lipid parameters and genotypes; (*ii*) Genotype frequency; and (*iii*) Serum lipid parameters: TC, TG, HDL-C and LDL-C.

### Data extraction

The data were abstracted using a standard method. The five investigators independently extracted data according to the author details and the following information was extracted from each study: first author's name, publication year, region, sample size, genotype information (number of genotypes, genotyping method), relationship between genotypes and serum lipid parameters. Discrepancies were resolved by discussion. When repeated publications of the same trial were identified, data were extracted from the repeated publications and reported as a single trial.

### Statistical analysis

Allele frequencies were determined by the allele counting method for each study. Relationships between continuous variables (lipid parameters) and genotypes were expressed as mean difference (MD) with 95% confidence intervals (CI). Pooled effects were calculated using a fixed effects model when there was no significant heterogeneity among the data from the contributing studies; however, a random effects model was used to ensure the robustness of the chosen model and susceptibility to outlier effects, or when there was significant heterogeneity among contributing study data, in which case a fixed effects model was used to ensure the robustness of the chosen model and examine susceptibility to outlier effects. Point estimates of MD were considered statistically significant when two-tailed *P*-values were ≤0.05. Heterogeneity was analysed using a chi-squared test and N − 1 degrees of freedom [41]. *I*² values of 25%, 50% and 75% correspond to low, medium and high levels of heterogeneity respectively. Sensitivity analyses were performed by omitting a single study at a time or by analysis using another model. We carried out statistical analysis by the Review Manager software 5.2.0. Stratified analysis was performed according to the ethnicity of participants.

## Results

### Characteristics of included studies

Seventy-four studies with 48,210 subjects [11–38,42–87] (Table S1), all published in English, met the inclusion criteria for this meta-analysis (Fig. S1). When the studies reported lipid parameter data for two different groups, we treated the groups as independent.

Thus, the meta-analysis of Pro12Ala (*PPARG*) included 67 eligible studies, containing 45,831 participants. According to ethnic origin, two Subgroups (Asian and non-Asian) were divided from each comparison. For Asian subgroup, Pro12Ala (*PPARG*) included 24 eligible studies and 40 separate comparisons of the levels of TC [17,18,20,21,23,24,26,28,29,31,38,46,47,55,57,59,61,63,64,67,70,71,79,81], 25 eligible studies and t 41 separate comparisons of the levels of TG [17,18,20,21,23,24,26,28,29,31,38,44,46,47,55,57,59,61,63,64,67,70,71,79,81], 22 eligible studies and 37 separate comparisons of the levels of HDL-C [17,18,21,23,24,26,28,29,31,44,46,47,55,57,61,63,64,67,70,71,79,81], and 17 eligible studies and 29 separate comparisons of the levels of LDL-C [17,18,23,24,28,31,46,47,55,57,63,64,67,70,71,79,81]. For non-Asian subgroup, Pro12Ala (*PPARG*) included 36 eligible studies and 45 separate comparisons of the levels of TC [11–15,19,22,25,27,36,37,48,50–54,56,62,65,66,68,69,72,74–78,80,82–87], 39 eligible studies and 50 separate comparisons of the levels of TG [11,13–16,19,22,25,27,36,37,39,48–54,56,58,60,62,65,66,68,69,72,74–78,80,82–85,87], 40 eligible studies and 50 separate comparisons of the levels of HDL-C [11–13,15,16,19,22,25,27,36,37,39,48–54,56,58,60,62,66,68,69,72–78,80,82–87], and 28 eligible studies and 37 separate comparisons of the levels of LDL-C [11–13,15,19,22,25,36,37,39,48,51–54,56,62,66,69,72–76,78,80,85,87].

The meta-analysis of C161T (*PPARG*) included 11 eligible studies, containing 3518 participants. Subgroup analysis was performed based on ethnicity (Asian and non-Asian). In Asian group, C161T (*PPARG*) included 6 eligible studies and 11 separate comparisons of the levels of TC [28,33–35,42,45], 5 eligible studies and 9 separate comparisons of the levels of LDL-C [28,33,35,42,45], 8 eligible studies and 13 separate comparisons of the levels of TG and HDL-C [28,33–35,42–45] respectively. In non-Asian group, C161T (*PPARG*) included 3 eligible studies and 6 separate comparisons of the levels of TC, TG, HDL-C and LDL-C respectively [15,25,32].

The meta-analysis of C1431T (*PPARG*) included 4 eligible studies, containing 5604 all Asian participants. A total of 3 eligible studies and 6 separate comparisons contained the levels of TC, LDL-C respectively [17,30,31]. A total of 4 eligible studies and 7 separate comparisons contained the levels of TG, HDL-C respectively [17,29–31].

### Associations with lipid levels

**Fig. 1:**
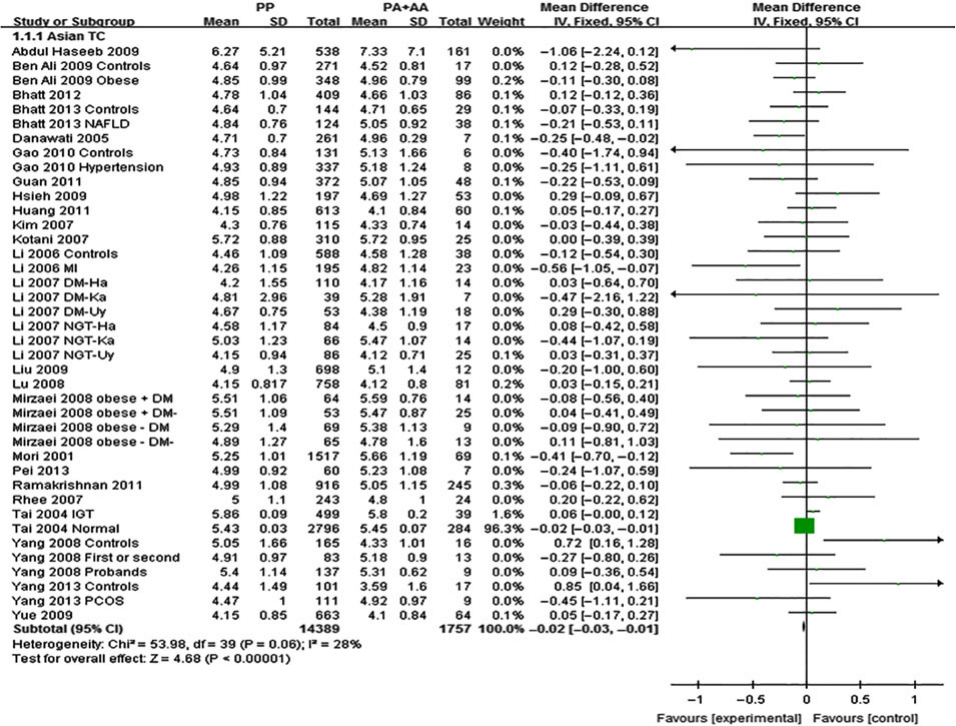
Forest plot of the association between *PPARG* Pro12Ala polymorphism and TC levels in Asian population (genetic model: PP *versus* PA + AA).

**Fig. 2:**
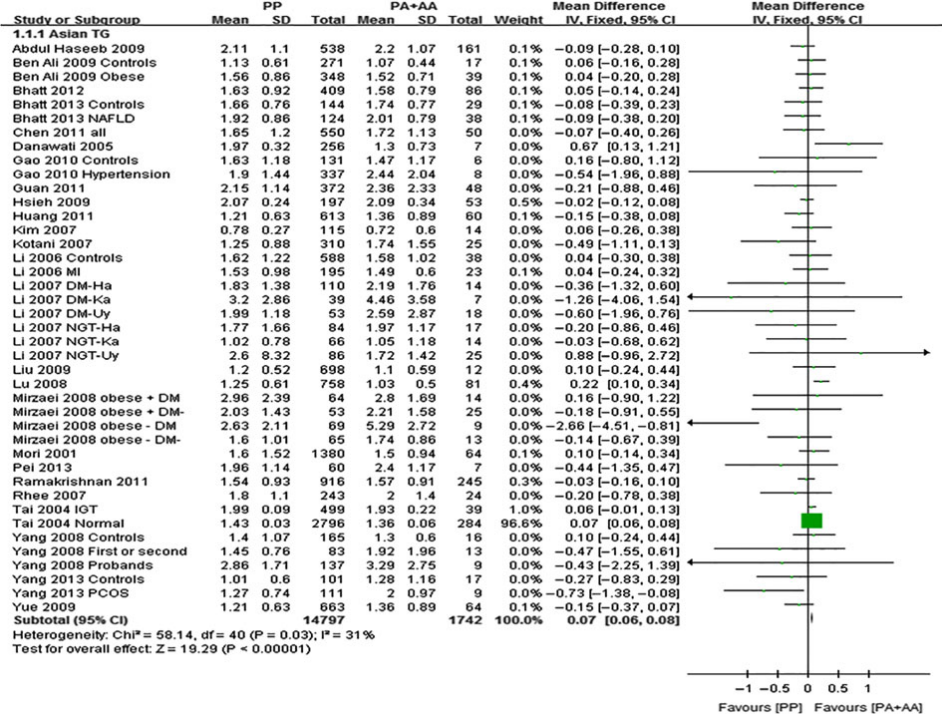
Forest plot of the association between *PPARG* Pro12Ala polymorphism and TG levels in Asian population (genetic model: PP *versus* PA + AA).

**Fig. 3:**
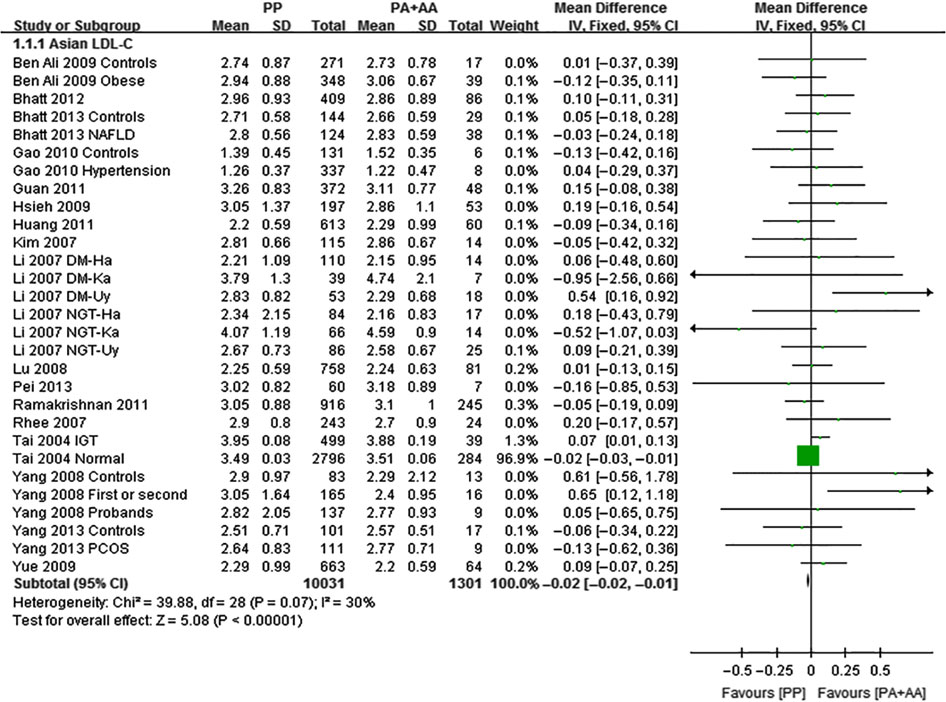
Forest plot of the association between *PPARG* Pro12Ala polymorphism and LDL-C levels in Asian population (genetic model: PP *versus* PA + AA).

The outcomes of Pro12Ala (*PPARG*) in Asian population: Pooling of data from eligible compared groups indicated that the group with the ‘PP’ (C/C genotype) genotype group had lower levels of TC (MD: −0.02, 95% CI: −0.03 to −0.01, *P* < 0.00001; *I*^*2*^ = 28%), LDL-C (MD: −0.02, 95% CI: −0.02 to −0.01, *P* < 0.00001; *I*^*2*^ = 30%) and higher levels of TG (MD: 0.06, 95% CI: 0.01–0.11, *P* < 0.00001; *I*^*2*^ = 30%) than the combined ‘PA+AA’ (PA = C/G genotype, AA = G/G genotype) genotype group. No statistically significant difference in the levels of HDL-C (MD: −0.01, 95% CI: −0.03 to 0.01, *P* = 0.54; *I*^*2*^ = 62%) was detected between the ‘PP’ and ‘PA+AA’ groups (Figs 1–3, Fig. S2).

**Fig. 4:**
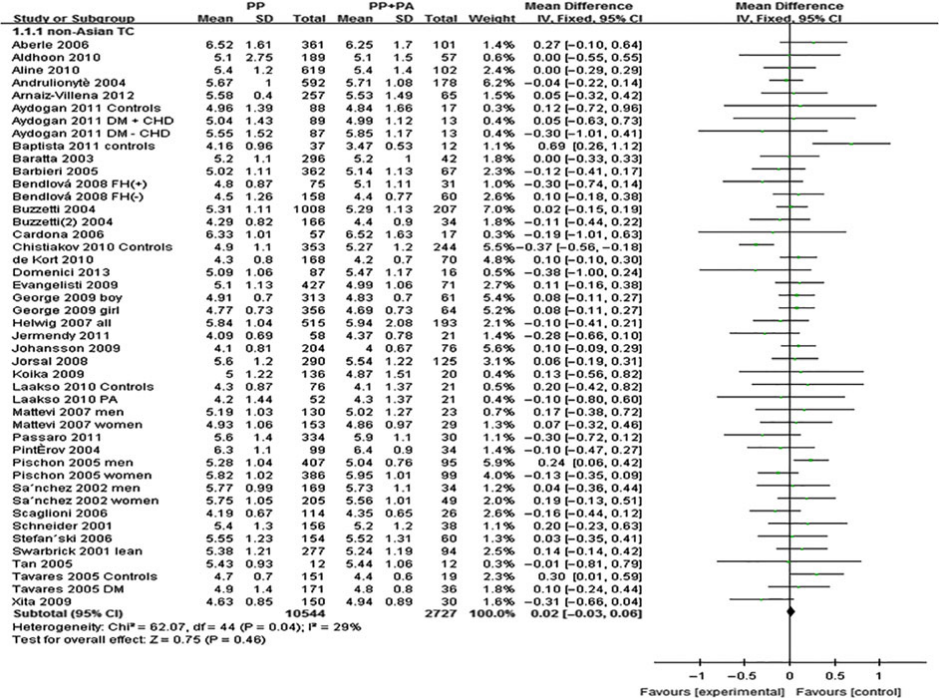
Forest plot of the association between *PPARG* Pro12Ala polymorphism and TC levels in non-Asian population (genetic model: PP *versus* PA + AA).

**Fig. 5:**
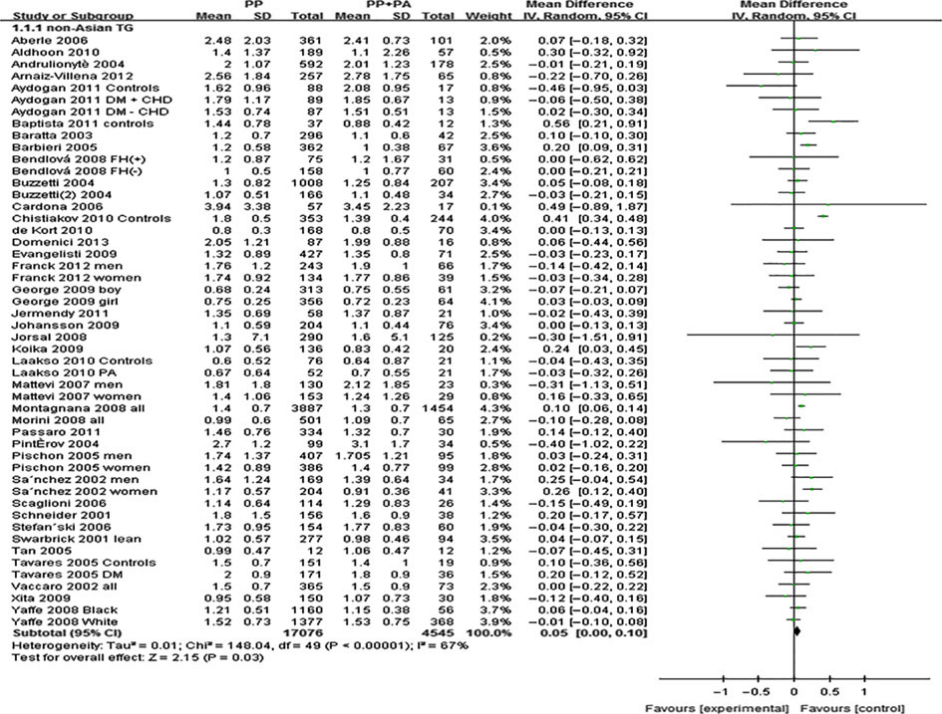
Forest plot of the association between *PPARG* Pro12Ala polymorphism and TG levels in non-Asian population (genetic model: PP *versus* PA + AA).

**Fig. 6:**
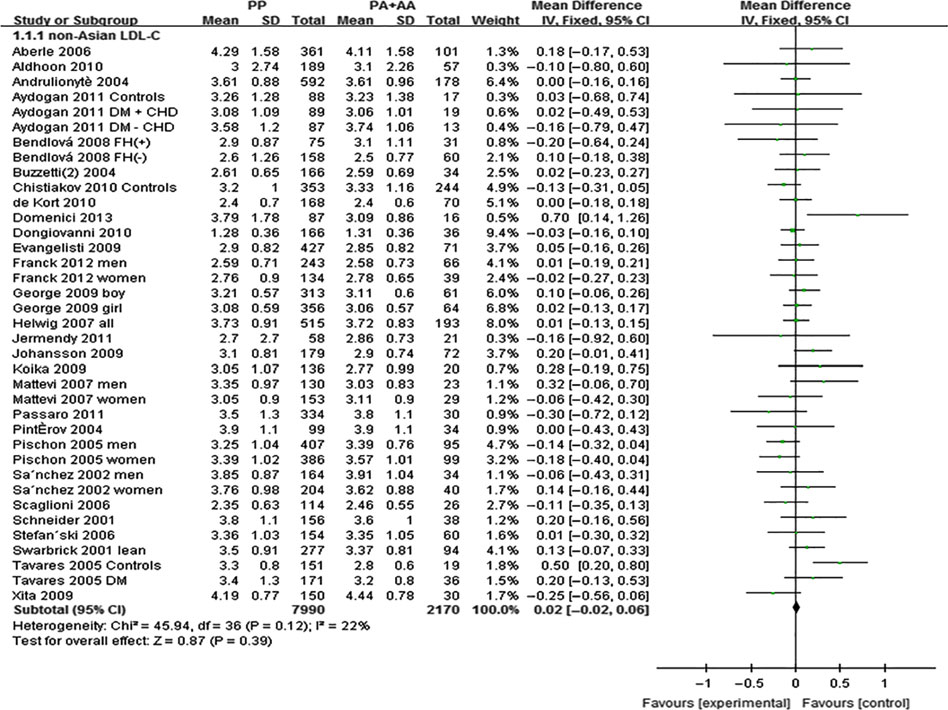
Forest plot of the association between *PPARG* Pro12Ala polymorphism and LDL-C levels in non-Asian population (genetic model: PP *versus* PA + AA).

The outcomes of Pro12Ala (*PPARG*) in non-Asian population: Pooling of data from eligible compared groups indicated that the group with the ‘PP’ genotype had higher levels of TG (MD: 0.06, 95% CI: 0.01–0.11, *P* = 0.02; *I*^*2*^ = 67%) than the combined ‘PA+AA’ genotype group. No statistically significant differences in the levels of TC (MD: 0.02, 95% CI: −0.03 to 0.06, *P* = 0.46; *I*^*2*^ = 29%), LDL-C (MD: 0.02, 95% CI: −0.02 to 0.06, *P* = 0.39; *I*^*2*^ = 22%) and HDL-C (MD: −0.01, 95% CI: −0.02 to 0.00, *P* = 0.06; *I*^*2*^ = 43%) were detected between the ‘PP’ and ‘PA+AA’ groups (Figs 4–6, Fig S3).

**Fig. 7:**
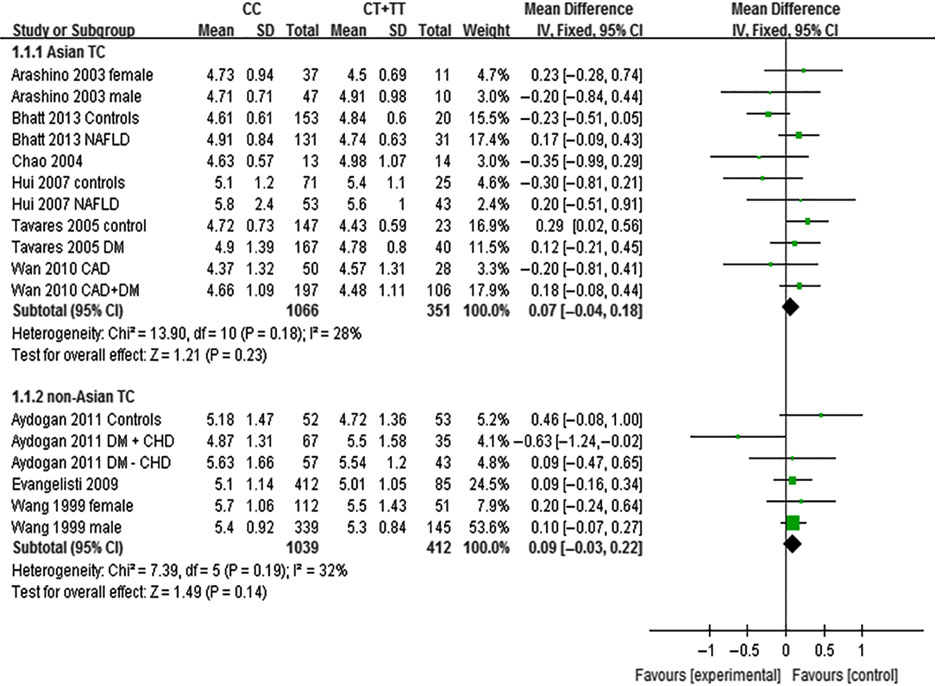
Forest plot of the association between *PPARG* C161T polymorphism and TC levels in Asian and non-Asian populations (genetic model: CC *versus* CT + TT).

**Fig. 8:**
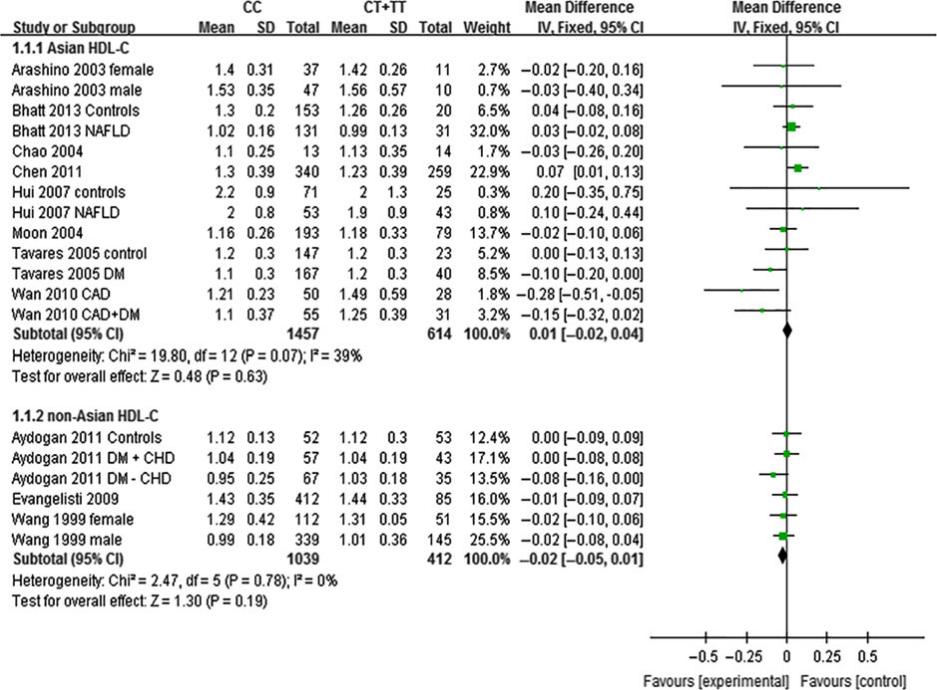
Forest plot of the association between *PPARG* C161T polymorphism and HDL-C levels in Asian and non-Asian populations (genetic model: CC *versus* CT + TT).

**Fig. 9:**
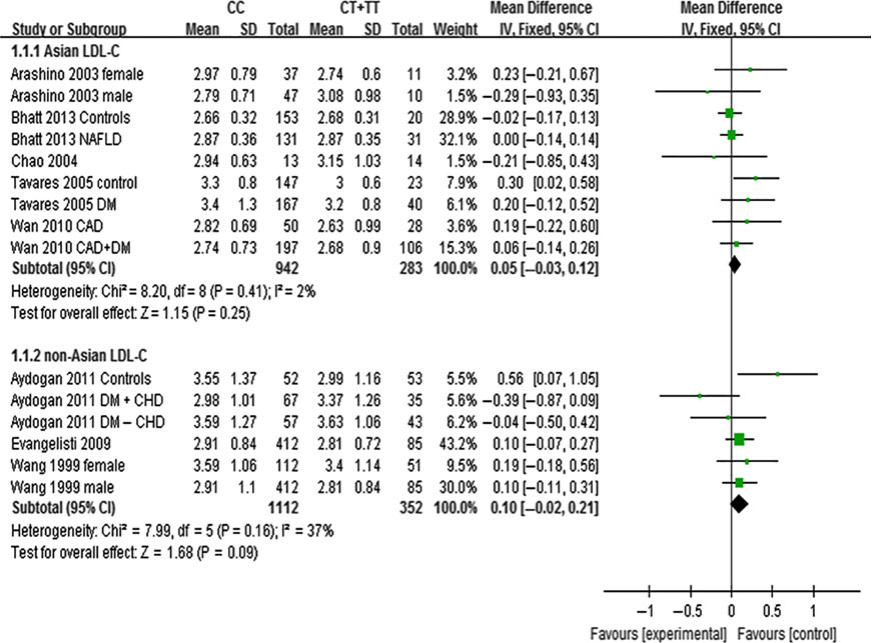
Forest plot of the association between *PPARG* C161T polymorphism and LDL-C levels in Asian and non-Asian populations (genetic model: CC *versus* CT + TT).

The outcomes of C161T (*PPARG*) in Asian population: In the subgroup analysis by ethnicity of study population, no statistically significant differences were detected in the levels of TC (MD: 0.07, 95% CI: −0.04 to 0.18, *P* = 0.23; *I*^*2*^ = 28%), TG (MD: 0.09, 95% CI: −0.06 to 0.25, *P* = 0.24; *I*^*2*^ = 69%), HDL-C (MD: 0.01, 95% CI: −0.02 to 0.04, *P* = 0.63; *I*^*2*^ = 39%) and LDL-C (MD: 0.05, 95% CI: −0.03 to 0.12, *P* = 0.25; *I*^*2*^ = 2%) between ‘CC’ (C/C genotype) and ‘CT + TT’ (C/T + T/T genotype) groups (Figs 7–9, Fig. S4).

The outcomes of C161T (*PPARG*) in non-Asian population: No statistically significant differences were detected in the levels of TC (MD: 0.09, 95% CI: −0.03 to 0.22, *P* = 0.14; *I*^*2*^ = 32%), TG (MD: 0.03, 95% CI: −0.09 to 0.16, *P* = 0.60; *I*^*2*^ = 30%), HDL-C (MD: −0.02, 95% CI: −0.05 to 0.01, *P* = 0.19; *I*^*2*^ = 0%) and LDL-C (MD: 0.10, 95% CI: −0.02 to 0.21, *P* = 0.09; *I*^*2*^ = 37%) in non-Asian populations between ‘CC’ and ‘CT +TT’ groups (Figs 7–9, Fig. S4).

**Fig. 10:**
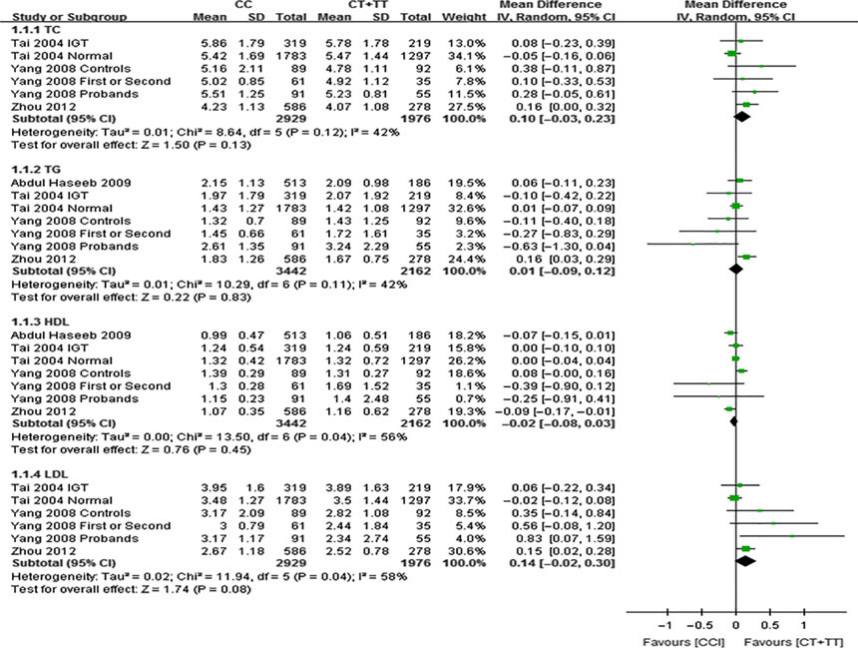
Forest plot of the association between *PPARG* C1431T polymorphism and lipid levels in Asian and non-Asian populations (genetic model: CC *versus* CT + TT).

The outcomes of C1431T (*PPARG*): all the study population were Asian. As shown in Figure 10, no significant differences in the levels of TC (MD: 0.1, 95% CI: −0.03 to 0.23, *P* = 0.13, *I*^*2*^ = 42%), TG (MD: 0.01, 95% CI: −0.09 to 0.12, *P* = 0.83, *I*^*2*^ = 42%), HDL-C (MD: −0.02, 95% CI: −0.08 to 0.03, *P* = 0.45, *I*^*2*^ = 56%), LDL-C (MD: 0.14, 95% CI: −0.02 to 0.30, *P* = 0.08, *I*^*2*^ = 0.08%) were detected between the CC and CT +TT groups.

### Sensitivity analysis

Sensitivity analyses were performed to assess the contribution of each study to the pooled estimate and by excluding individual studies one at a time and recalculating the pooled MD estimates for the remaining studies, and we found that this did not lead to a substantial alteration in the corresponding pooled MD. Eliminating the studies with average years more than 75 or less than 18 did not substantially change the pooled point estimate. What is more, performing transition of model also did not substantially change the pooled point estimates for any of the observed associations.

### Heterogeneity Analysis

For most of the outcomes of serum lipid levels, the *I*^*2*^ values of heterogeneity were lower than 50%. Only the levels of heterogeneity at Pro12Ala (Asian: HDL-C and non-Asian: TG), C161T (Asian: TG) and C1431T (Asian: LDL-C) were medium. To explore the sources of heterogeneity, we performed subgroup analyses with stratification by case and control status; heterogeneity still existed, but the corresponding pooled MD was not substantially altered.

## Discussion

Cardiovascular disease, a kind of chronic disease with high prevalence and morbidity, has attracted more and more researchers to study its related fields throughout the world [88]. Previous researches have revealed that dyslipidaemia is closely related to the occurrence and progress of CVD. Various candidate genes have been reported as predisposing factors of dyslipidaemia, including those involved in lipid transport and metabolism. *PPARG* as a member of the nuclear receptor superfamily regulates adipocyte differentiation, adipocyte-specific gene expression and insulin action. Recently, an increasing number of studies have been carried out to determine the association between the difference *PPARG* loci polymorphisms and serum lipid levels, but the results are inconclusive. Li *et al*. [38] concluded that ‘PA+AA’ subjects had lower levels of HDL-C and a trend towards higher levels of TG, LDL-C compared with ‘PP’ subjects, whereas, Gonzalez Sanchez *et al*. [36] reported that the Ala12 allele was associated with lower TG levels. What is more, no relation between the polymorphism and the levels of TC, HDL-C and LDL-C or TG could be detected by some investigators [37,62,79]. Several studies did not find the associations between C161T polymorphism and serum lipid levels in different population [44,45]. However, Bhatt *et al*. [28] tended to believe that ‘TT’ genotype group have higher levels of TG, TC than ‘CT + TT’ genotype group in C161T polymorphism. For C1431T, T-allele carriers had found increasing HDL-C levels [30,31]. These inconsistent results may be because of a small sample size.

As the results in our meta-analysis showed, at Pro12Ala (*PPARG*), the group with the ‘PP’ genotype had lower levels of TC, LDL-C and higher levels of TG than the combined ‘PA+AA’ genotype group in Asian population, and the group with the ‘PP’ genotype had higher levels of TG than the combined ‘PA+AA’ genotype group in non-Asian population. No statistically significant differences in the levels of TC, TG, HDL-C, LDL-C were detected between different genotypes in C161T (Asian or non-Asian) and C1431T (Asian) polymorphisms.

It is the first time that meta-analysis was conducted to explore the association between *PPARG* polymorphisms and serum lipid levels. In this study, we performed a meta-analysis of 74 studies investigating such associations. In addition, for the majority of pooled effects, calculated heterogeneity was low. Part of MD values for associations with the total of lipid parameters (*P* < 0.00001) indicated very significant effects, including TC, TG and LDL-C levels with Pro12Ala (*PPARG*) in Asian population, which demonstrate that the conclusions in our meta-analysis are robust.

High heterogeneity is a potential problem that may affect the interpretation of the results. But heterogeneity analysis showed that the *I*^*2*^ values of heterogeneity in most of our outcomes were lower than 50%. Only the levels of heterogeneity in the outcomes of Pro12Ala (Asian: HDL-C and non-Asian: TG), C161T (Asian: TG) and C1431T (Asian: LDL-C) were medium. These indicate the reliability of the results in our meta-analysis. The medium heterogeneity may be because different groups of the included studies had different genetic backgrounds and environmental factors. It is well known that both serum lipid levels and CVD are affected by genetic and environmental factors, such as dietary patterns, lifestyle, obesity, physical inactivity.

For better interpreting the results, some limitations of this meta-analysis should be acknowledged. Firstly, this meta-analysis focused only on papers published in English and the ones that reported in other languages may bias the present results. Secondly, we did not perform subgroup analysis by the factors such as smoking habits, diet pattern, alcohol use, case/control because insufficient data could be extracted. In addition, gene–gene and gene–environment interactions should also be considered in the analysis. However, heterogeneity in most of our outcomes was low, so that these limitations do not affect the reliability of the results in our meta-analysis.

Thus, it is necessary to conduct a study using standardized unbiased methods. Moreover, gene–gene and gene–environment interactions should also be considered in the analysis. Such studies taking these factors into account may eventually lead to better, more comprehensive understanding of the association between the common polymorphisms of the *PPARG* and serum lipid levels.

## Conclusion

This meta-analysis was a renewed and confirmed study to assess the association between common polymorphisms in *PPARG* and serum lipid levels. As the results in our meta-analysis showed, there is a prominent association between Pro12Ala polymorphism and the levels of TC, LDL-C and TG in the Asian population. No statistically significant differences in the levels of TC, TG, HDL-C, LDL-C were detected between different genotypes in C161T (Asian or non-Asian population) and C1431T (Asian population) polymorphisms.

## Supporting information

**Figure S1** Flow chart showing study selection process. **Figure S2** Forest plot of the association between *PPARG* Pro12Ala polymorphism and HDL-C levels in Asian population (genetic model: PP *versus* PA + AA). **Figure S3** Forest plot of the association between *PPARG* Pro12Ala polymorphism and HDL-C levels in non-Asian population (genetic model: PP *versus* PA + AA). **Figure S4** Forest plot of the association between *PPARG* C161T polymorphism and TG levels in Asian and non-Asian populations (genetic model: CC *versus* CT + TT). **Table S1** Baseline characteristics of included studies.
