# Supplementary material for: Association of the variants in the PPARG gene and serum lipid levels: a meta-analysis of 74 studies
Source: J Cell Mol Med. 2014 Sep 30;19(1):198–209. doi: 10.1111/jcmm.12417 (PMC4288363; doi:10.1111/jcmm.12417)
Supplement: Supplementary file 1 [file jcmm0019-0198-sd1.docx]

**Supplemental Table 1. Baseline characteristics of included studies**

| SNP | First author | Year | Country | Age(years) | SS(M/F) | SOC |  | | GM | |
| --- | --- | --- | --- | --- | --- | --- | --- | --- | --- | --- |
| Pro12Ala |  |  |  |  |  |  |  | |  | |
|  | Jermendy [22] | 2011 | Hungary | 13.8 ± 2.7 | 79 (39/40) | Hospital-based | | PCR-RFLP | |  |
|  | Mirzaei [61] | 2009 | Iran | 45.0 ± 13.87 | 312 (149/129) | Population-based | | PCR-RFLP | |  |
|  | Vaccaro [49] | 2002 | Italy | 45.6 ± 6 | 438 (315/123) | Population-based | | PCR-RFLP | |  |
|  | Li [64] | 2007 | China | 51.3 ± 15.1 | 534 (-/-) | Population-based | | PCR-RFLP | |  |
|  | Johansson [69] | 2009 | Europe | - | 285 (122/163) | Hospital-based | | Taqman | |  |
|  | Barbieri [14] | 2005 | Italy | 72 ± 26 | 429 (150/279) | Population-based | | PCR-RFLP | |  |
|  | Montagnana [60] | 2008 | Europe | 57.6 ± 5.9 | 5341 (4176/1165) | Hospital-based | | Taqman | |  |
|  | Swarbrick [51] | 2001 | Australia | 53.0 ± 12.8 | 663 (325/338) | Hospital-based | | - | |  |
|  | Sa´nchez [36] | 2002 | Spain | 49 ± 8 | 464 (210/254) | Population-based | | PCR-RFLP | |  |
|  | PintÈrova [37] | 2004 | Czech | 65.3 ± 9.6 | 133 (-/-) | Population-based | | PCR-RFLP | |  |
|  | Tan [50] | 2005 | England | - | 774 (550/524) | Population-based | | - | |  |
|  | Aberle [13] | 2006 | German | 43.9 ± 13.7 | 462 (205/257) | Hospital-based | | - | |  |
|  | Cardona [27] | 2006 | Spain | 43.5 ± 11 | 74 (57/17) | Hospital-based | | PCR-RFLP | |  |
|  | Yaffe [16] | 2008 | America | 73.6 ± 2.8 | 2961 (1524/1437) | Population-based | | PCR-RFLP | |  |
|  | Rhee [55] | 2007 | Korea | 58.2 ± 10.8 | 267 (158/109) | Hospital-based | | Taqman | |  |
|  | Mattevi [62] | 2007 | Brazil | 39.9 ± 31.0 | 335 (153/182) | Population-based | | PCR-RFLP | |  |
|  | Helwig [12] | 2007 | German | 58.7 ±5.6 | 708 (708/0) | Population-based | | Taqman | |  |
|  | Kim [67] | 2007 | Korea | 42.71 ± 8.56 | 129 (0/129) | Population-based | | PCR-RFLP | |  |
|  | Bendlová [80] | 2008 | Czech | 32 ± 11 | 324 (99/225) | - |  | | SSCP | |
|  | Lu [63] | 2008 | China | 94.6 ± 4.0 | 839 (271/568) | Population-based | | PCR-RFLP | |  |
|  | Yang [31] | 2008 | China | 51.1 ± 15.7 | 423 (192/231) | Hospital-based | | PCR-RFLP | |  |
|  | Evangelisti [25] | 2009 | Italy | - | 497 (347/150) | Hospital-based | | PCR-RFLP | |  |
|  | Ben Ali [81] | 2009 | Tunisia | 45.77 ± 11.5 | 675 (319/356) | Hospital-based | | PCR-RFLP | |  |
|  | Xita [48] | 2009 | Greece | 23.7 ± 6.4 | 180 (-/180) | Hospital-based | | PCR-RFLP | |  |
|  | de Kort [75] | 2010 | Netherlands | - | 238 (124/114) | - |  | | Taqman | |
|  | Chistiakov [76] | 2010 | Russia | 60.95 ± 9.00 | 1165 (515/670) | Hospital-based | | Taqman | |  |
|  | Gao [24] | 2010 | China | 53.33 ± 16.00 | 482 (330/152) | Hospital-based | | Direct sequencing | |  |
|  | Ramakrishnan [18] | 2011 | Indian | - | 1161 (-) | Population-based | | PCR-RFLP | |  |
|  | Aydogan [15] | 2011 | Greece | 57.78 ± 11.92 | 307 (160/147) | Hospital-based | | PCR-RFLP | |  |
|  | Passaro [19] | 2011 | Italy | 55.75 ± 12.80 | 364 (-) | Hospital-based | | PCR-RFLP | |  |
|  | Bhatt [79] | 2012 | India | 39.48 ± 8.83 | 495 (276/219) | Hospital-based | | PCR-RFLP | |  |
|  | Yang [47] | 2013 | China | 27.86 ± 6.17 | 238 (238/-) | Hospital-based | | PCR-RFLP | |  |
|  | Domenici [74] | 2013 | Brazil | 44.5 ± 2.5 | 103 (47/56) | Hospital-based | | PCR-RFLP | |  |
|  | Abdul-Haseeb [29] | 2009 | India | 61.85 ± 10.84 | 699 (425/274) | Hospital-based | | PCR-RFLP | |  |
|  | Arnaiz-Villena [84] | 2012 | Spain | 38.15 ± 9.30 | 322 (193/129) | Population-based | | Direct sequencing | |  |
|  | Aldhoon [87] | 2010 | Czech | 49.0 ± 11.9 | 246 (0/246) | Hospital-based | | Taqman | |  |
|  | Chen [44] | 2011 | China | 46.3 ±11.4 | 600 (309/291) | Hospital-based | | Taqman | |  |
|  | Bhatt [28] | 2013 | India | 37.63 ± 6.97 | 335 (-/-) | Hospital-based | | PCR-RFLP | |  |
|  | Stefan´ski [52] | 2006 | Poland | 64.2 ± 8.4 | 214 (95/119) | Hospital-based | | PCR-RFLP | |  |
|  | Koika [66] | 2009 | Greece | 22.46 ± 4.41 | 156 (0/156) | Hospital-based | | PCR-RFLP | |  |
|  | Andrulionytè [85] | 2004 | Europe | 54.7 ± 7.9 | 770 (387/383) | - |  | | PCR-RFLP | |
|  | Baratta [82] | 2003 | Italy | 37.88 ± 12.78 | 338 (148/190) | Hospital-based | | PCR-RFLP | |  |
|  | Tai [17] | 2004 | Asian | 38.57 ± 12.34 | 4038 (1869/2,169) | Population-based | | - | |  |
|  | Dongiovanni [73] | 2010 | Finland | 47.4 ± 11.00 | 202 (161/41) | Hospital-based | | - | |  |
|  | Pischon [56] | 2005 | American | 62.77 ± 8.00 | 987 (502/485) | Hospital-based | | PCR-RFLP | |  |
|  | Franck [39] | 2012 | Sweden | - | 482 (309/173) | Hospital-based | | Taqman | |  |
|  | Yue [46] | 2009 | China | 93.5 ± 3.35 | 697 (-) | Population-based | | Taqman | |  |
|  | Liu [20] | 2009 | China | 64.37 ± 11.94 | 760 (382/378) | Hospital-based | | PCR-RFLP | |  |
|  | Buzzetti [77] | 2004 | Italy | 42.65 ± 13.55 | 1215 (366/849) | Population-based | | PCR-RFLP | |  |
|  | Jorsal [68] | 2008 | Denmark | 42.27 ± 10.53 | 415 (252/163) | Hospital-based | | PCR-RFLP | |  |
|  | Mori [59] | 2001 | Japan | 60.85 ± 11.72 | 2201 (995/1206) | - |  | | Taqman | |
|  | Schneider [53] | 2001 | German | 61.80 ± 9.82 | 194 (194/0) | - |  | | - | |
|  | Baptista [83] | 2011 | Columbia | 38.8 ± 11.2 | 49 (22/27) | Population-based | | PCR-RFLP | |  |
|  | Danawati [26] | 2005 | Indonesia | 58.00 ± 10.01 | 337 (158/179) | Hospital-based | | PCR-RFLP | |  |
|  | Tavares [11] | 2005 | Brazil | 53.55 ± 12.15 | 377 (148/229) | Hospital-based | | Direct sequencing | |  |
|  | Li [38] | 2006 | China | 60.73 ± 9.27 | 844 (506/338) | - |  | | PCR-RFLP | |
|  | George [72] | 2009 | Greece | 11.2 ± 0.7 | 794 (374/420) | Population-based | | iPLEX MassARRAY | |  |
|  | Guan [71] | 2011 | China | 44.19 ± 13.06 | 420 (357/63) | Hospital-based | | PCR-RFLP | |  |
|  | Hsieh [23] | 2009 | China | - | 250 (119/131) | Hospital-based | | PCR-RFLP | |  |
|  | Morini [58] | 2008 | Italy | 36.59 ± 11.85 | 566 (211/355) | Population-based | | 3333 | |  |
|  | Aline [86] | 2010 | Brazil | 57.71 ± 9.92 | 721 (347/374) | Hospital-based | | TaqMan | |  |
|  | Buzzetti [78] | 2004 | Italy | 10.38 ± 2.8 | 200 (95/105) | Hospital-based | | TaqMan | |  |
|  | Scaglioni [54] | 2006 | Italy | 10.2 ± 2.7 | 140 (82/58) | Hospital-based | | TaqMan | |  |
|  | Laakso [65] | 2010 | Finland | 7.5 ± 0.9 | 170 (28/142) | Hospital-based | | TaqMan | |  |
|  | Kotani [21] | 2007 | Japan | 52.57 ± 12.63 | 335 (0/335) | Population-based | | PCR-RFLP | |  |
|  | Pei [57] | 2013 | China | 56.63 ± 8.59 | 67 (39/28) | Hospital-based | | MALDI-TOF | |  |
|  | Huang [70] | 2011 | China | 93.54 ± 3.36 | 673 (221/452) | Population-based | | PCR-RFLP | |  |
| C161T |  |  |  |  |  |  |  | |  | |
|  | Tavares [33] | 2005 | Brazil | 53.55 ± 12.15 | 377 (148/229) | Hospital-based | | Direct sequencing | |  |
|  | Arashino [45] | 2003 | Japan | 9.5 ± 0.72 | 105 (57/48) | Hospital-based | | PCR-RFLP | |  |
|  | Hui [34] | 2007 | China | 50.5 ± 11.39 | 192 (80/112) | Population-based | | PCR-RFLP | |  |
|  | Chao [35] | 2004 | China | 31.67 ± 5.16 | 27 (-/-) | Hospital-based | | PCR-RFLP | |  |
|  | Wan [42] | 2010 | China | - | 467 (-/-) | Hospital-based | | PCR-RFLP | |  |
|  | Moon [43] | 2004 | Korea | 66.00 ± 9.61 | 272 (-/-) | Hospital-based | | Genetic Analyser | |  |
|  | Chen [44] | 2011 | China | 46.3 ± 11.4 | 600 (309/291) | Hospital-based | | Taqman | |  |
|  | Bhatt [28] | 2013 | India | 37.63 ± 6.97 | 335 (-/-) | Hospital-based | | PCR-RFLP | |  |
|  | Wang [32] | 1999 | Italy | - | 566 (211/355) | - |  | | - | |
|  | Evangelisti [25] | 2009 | Italy | - | 497 (347/150) | Hospital-based | | PCR-RFLP | |  |
|  | Aydogan [15] | 2011 | Greece | 57.78 ± 11.92 | 307 (160/147) | Hospital-based | | PCR-RFLP | |  |
| C1431T |  |  |  |  |  |  |  | |  | |
|  | Zhou [30] | 2012 | China | - | 1872 (1338/534) | Hospital-based | | Direct sequencing | |  |
|  | Tai [17] | 2004 | Asian | 38.57 ± 12.34 | 4038 (1869/2,169) | Hospital-based | | Taqman | |  |
|  | Yang [31] | 2008 | China | 51.1 ± 15.7 | 423 (192/231) | Hospital-based | | PCR-RFLP | |  |
|  | Abdul-Haseeb [29] | 2009 | India | 61.85 ± 10.84 | 699 (425/274) | Hospital-based | | PCR-RFLP | |  |

SS: sample size; M: male; F: female; SOC: source of population; GM: genotyping methods; PCR-RFLP: polymerase chain reaction restriction fragment length polymorphism; Taqman: real-time polymerase chain reaction. SSCP: Single Strand Conformation Polymorphism.

**Supplemental Figure 1. Flow chart showing study selection process.**


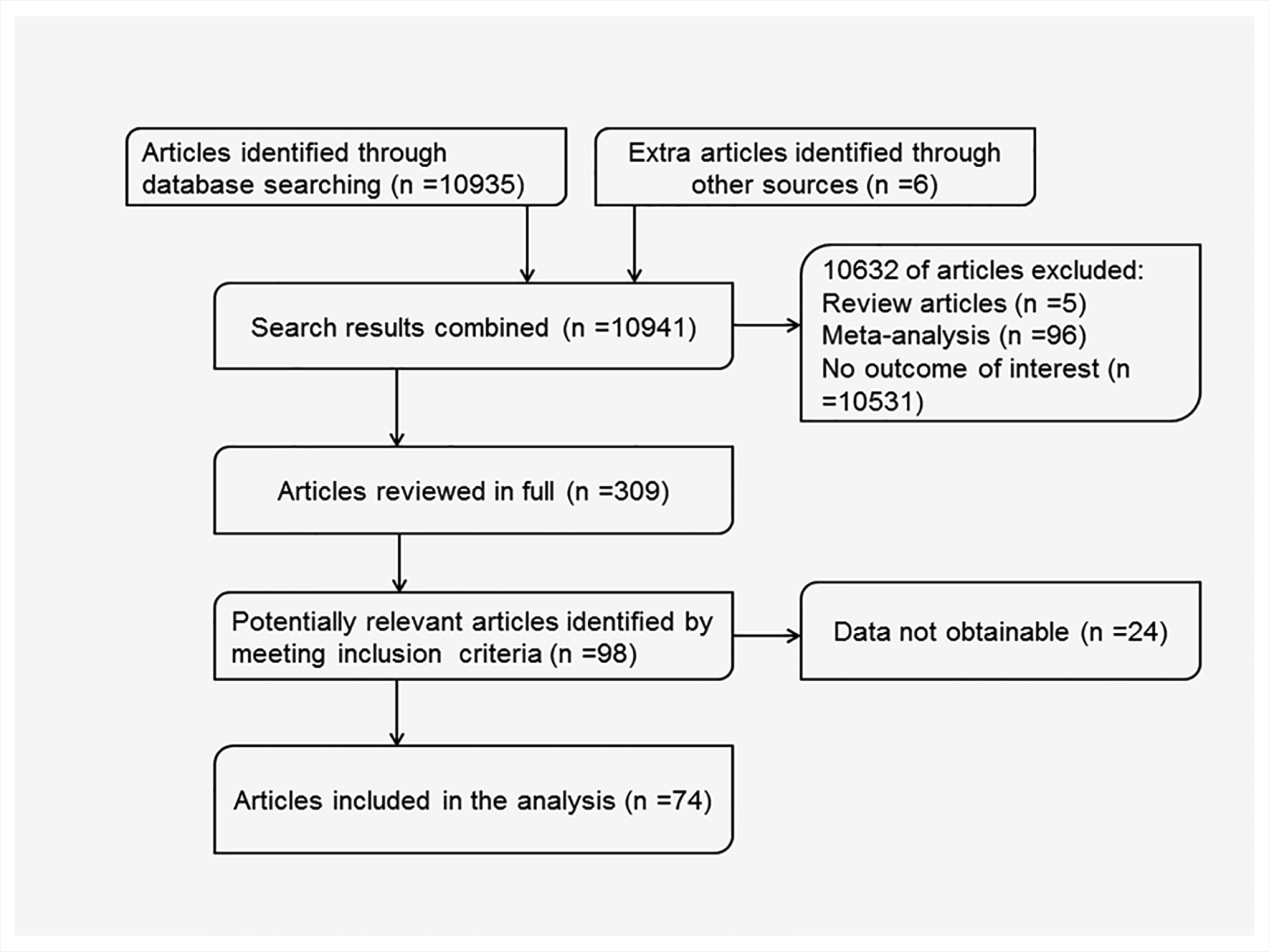


**Supplemental Figure 2. Forest plot of the association between *PPARG* Pro12Ala polymorphism and HDL-C levels in Asian population (genetic model: PP vs. PA + AA).**

**
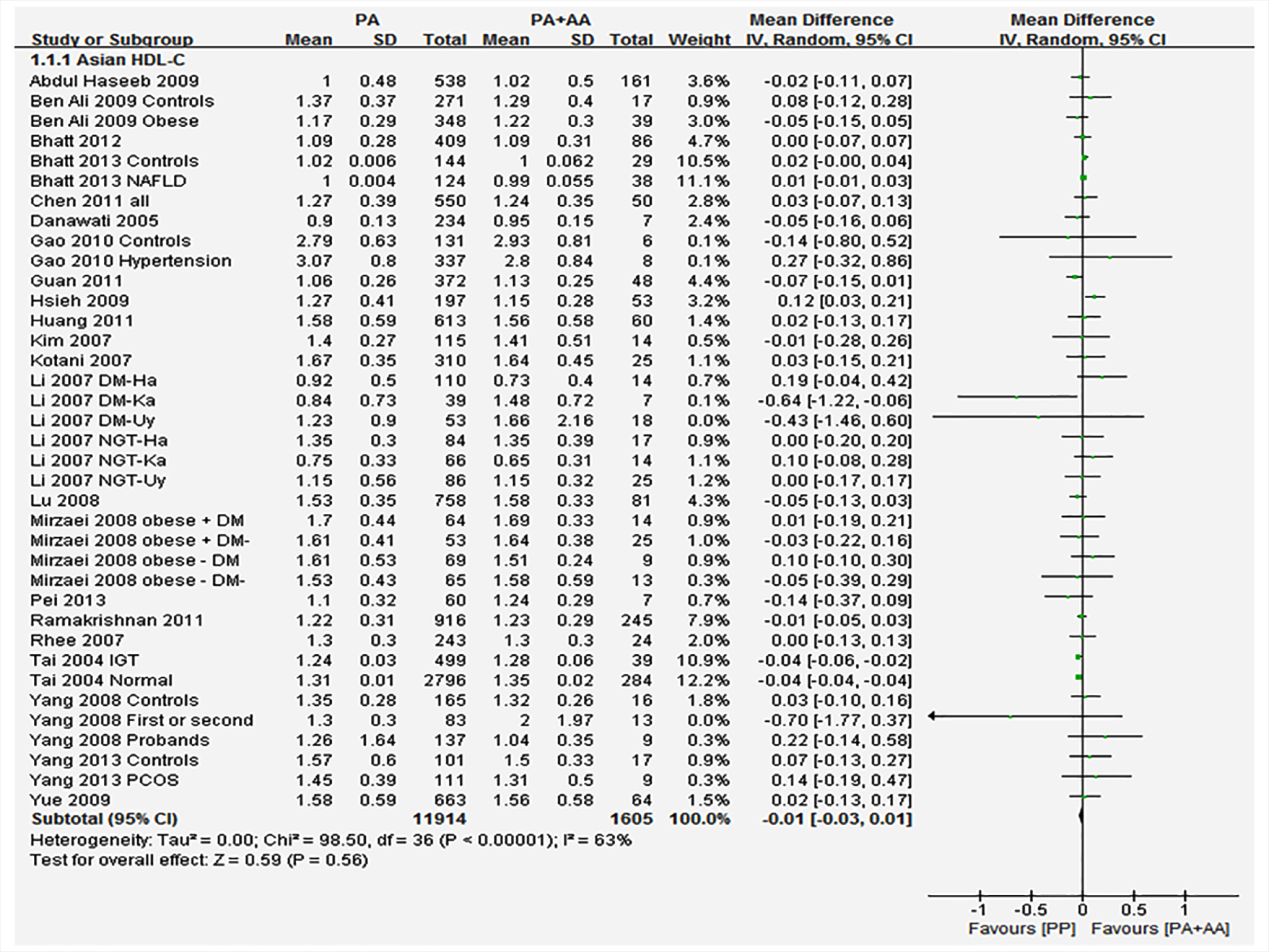
**

**Supplemental Figure 3. Forest plot of the association between *PPARG* Pro12Ala polymorphism and HDL-C levels in non-Asian population (genetic model: PP vs. PA + AA).**

**
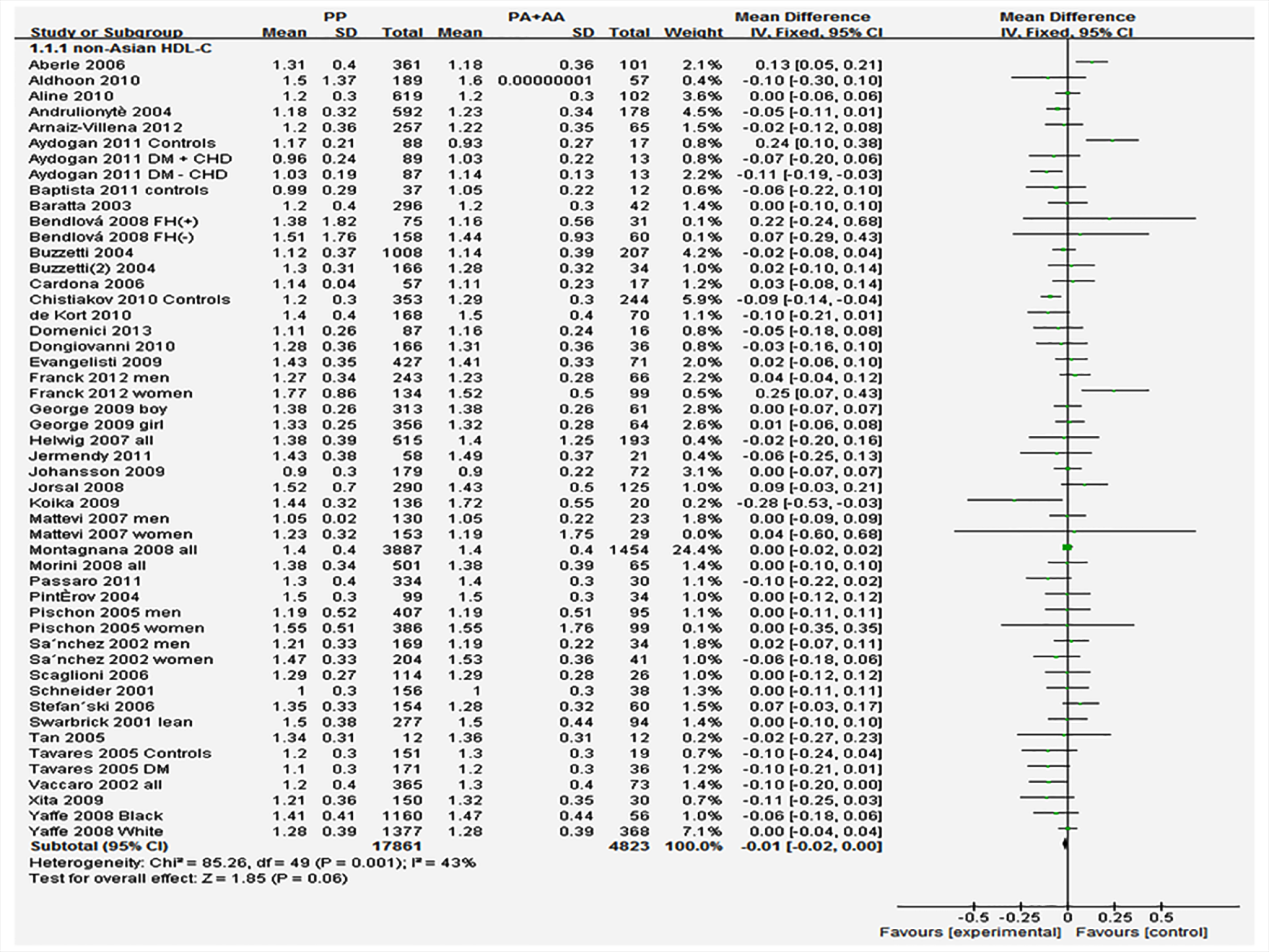
**

**Supplemental Figure 4. Forest plot of the association between *PPARG* C161T polymorphism and TG levels in Asian and non-Asian populations (genetic model: CC vs. CT + TT).**

**
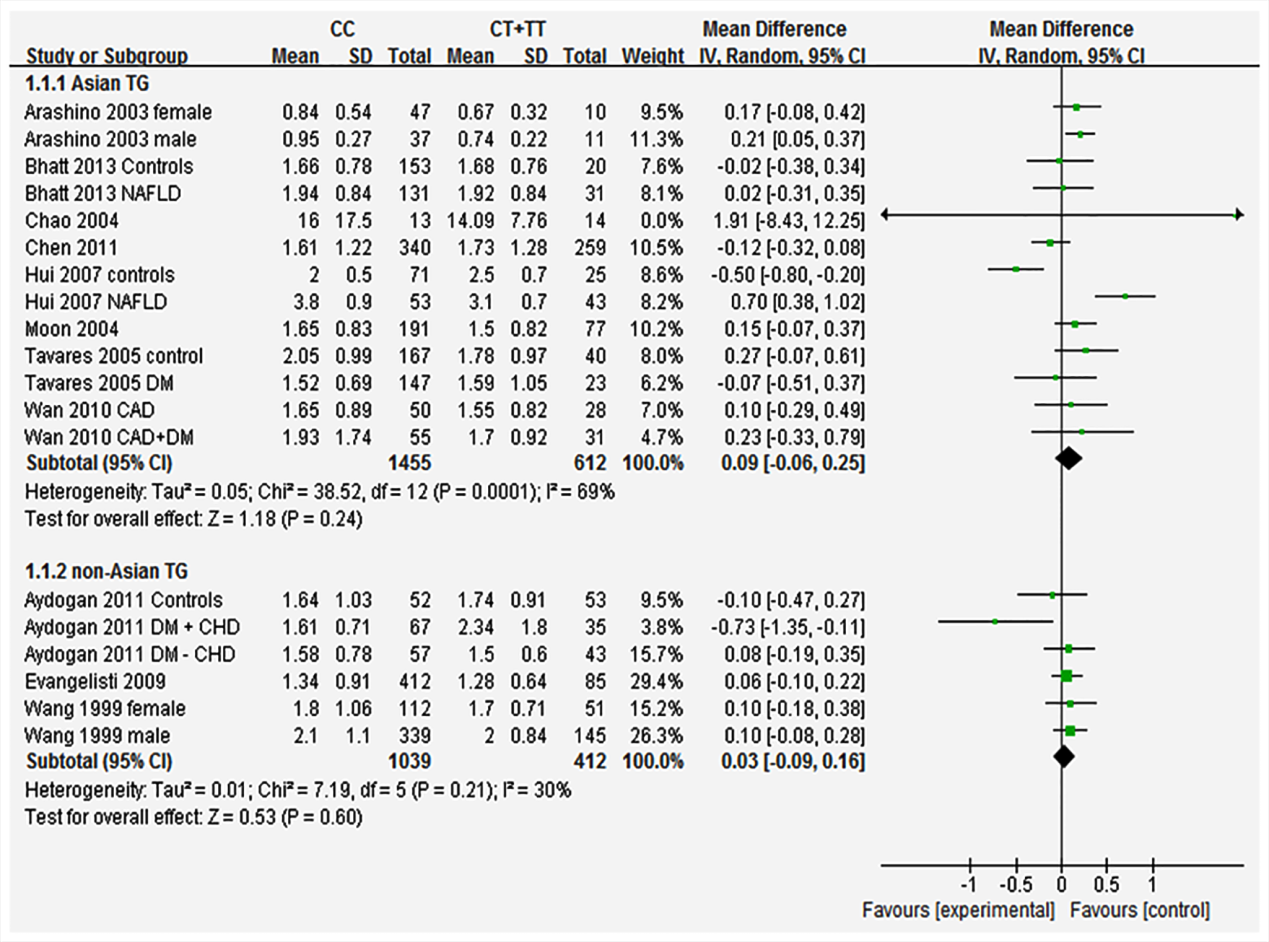
**
